# Impact of Platelet Transfusion on Survival of Patients with Intracerebral Hemorrhage after Administration of Anti-Platelet Agents at a Tertiary Emergency Center

**DOI:** 10.1371/journal.pone.0097328

**Published:** 2014-05-28

**Authors:** Yuhko Suzuki, Takao Kitahara, Kazui Soma, Shingo Konno, Kimitoshi Sato, Sachio Suzuki, Hidehiro Oka, Masaru Yamada, Kiyotaka Fujii, Yukio Kitahara, Yuji Yamamoto, Takashi Otsuka, Yoshihiro Sugiura, Yuhsaku Kanoh, Yoshiko Tamai, Hitoshi Ohto

**Affiliations:** 1 Departments of Blood Transfusion and Transplantation Immunology, Fukushima Medical University, Fukushima, Fukushima, Japan; 2 Departments of Emergency and Critical Care Medicine, Kitasato University School of Medicine, Sagamihara, Kanagawa, Japan; 3 Department of Neurosurgery, Kitasato University School of Medicine, Sagamihara, Kanagawa, Japan; 4 Department of Neurosurgery, Fuchinobe General Hospital, Sagamihara, Kanagawa, Japan; 5 Department of Neurosurgery, Ashigarakami Hospital, Ashigarakami, Kanagawa, Japan; 6 Department of Neurosurgery, Sagamihara-chuo Hospital, Sagamihara, Kanagawa, Japan; 7 Department of Neurology, Fukushima Medical University, Fukushima, Fukushima, Japan; 8 Department of Clinical Laboratory Medicine, Kitasato University School of Medicine, Sagamihara, Kanagawa, Japan; 9 Department of Transfusion, Hirosaki University School of Medicine, Hirosaki, Japan; German Red Cross Blood Service Frankfurt, Germany

## Abstract

This study examined the impact of platelet transfusion (PLT) on the survival of intracerebral hemorrhage (ICH) patients who had been administered anti-platelet agents (APA). This retrospective cohort analysis investigated 432 patients (259 men, 60%) who were newly diagnosed with ICH between January 2006 and June 2011 at the tertiary emergency center of Kitasato University Hospital. Median age on arrival was 67.0 years (range, 40–95 years). ICH was subcortical in 72 patients (16.7%), supratentorial in 233 (53.9%), and infratentorial in 133 (30.8%). PLT was performed in 16 patients (3.7%). Within 90 days after admission to the center, 178 patients (41.2%) had died due to ICH. Before the onset of ICH, 66 patients had been prescribed APA because of atherosclerotic diseases. Multivariate regression analysis indicated APA administration was an independent risk factor for death within 7 days (odds ratio, 5.12; P = 0.006) and within 90 days (hazard ratio, 1.87; P = 0.006) after arrival. Regarding the effect of a PLT in ICH patients with APA, no patient with PLT died. PLT had a survival benefit on patients with ICH, according to our analysis. Further prospective analysis is necessary to confirm the effects of PLT on survival in ICH with APA.

## Introduction

Recently, for secondary prevention of many kinds of coronary heart disease (CHD) and thrombotic diseases [1], [2], antithrombotic agents (ATA), including anti-platelet agents (APA) such as aspirin and anticoagulants such as warfarin, have been widely prescribed for patients, and sometimes two or more types of ATA are used concomitantly [1], [2], [3], [4]. Among patients receiving ATA, bleeding complications such as intracerebral hemorrhage (ICH) are becoming the issue of most concern.

The Japanese population shows a relatively high incidence of ICH according to the Hisayama [5] and Shibata [6] studies. According to reports from the Japanese Ministry of Health, Labour, and Welfare, cerebrovascular disease (including ICH, cerebral infarction, and subarachnoid hemorrhage) is the third most common cause of death in Japan. Furthermore, in Japan as well as other countries, ICH is among the major causes of stroke. For example, ICH is the second-most common cause of stroke in Italy [7], and is responsible for 15% of strokes reported in the USA [8].

With this background, increasing concern has emerged about the possibility of an ICH in patients receiving APA for a background chronic medical condition. The actual risk of ICH with APA is estimated as 0.2–0.3% per year [9]. Several articles have recently been published showing inferior prognosis of ICH patients who are taking APA compared to those without APA treatment [10], [11], [12], [13]. The predominant APAs are cyclooxygenase-1 inhibitors such as aspirin [14], [15] and anti-P2Y12 antagonists [16], [17], [18] such as clopidogrel and ticlopidine. The efficacy of APA varies depending on the genetic background of the patient [15], [19], [20]. However, to date, laboratory examinations have not been routinely and widely applied to check the effects of APA. To recover the platelet functions inhibited by APA, supplying platelets with normal function in the form of platelet transfusion (PLT) might seem efficacious. Although several reports have described the effects of a PLT on survival of ICH patients who are taking APA, nevertheless the effect of a PLT on the outcome of ICH still remains unclear [10], [21], [22], [23], [24], [25]. For example, Creutzfeldt et al reported there was no clear benefit in terms of survival in the administration of a PLT to ICH patients taking APA [10] and Ducruet et al reported that a PLT did not reduce the frequency of hematoma expansion in ICH patients receiving APA [23]. In contrast, Naidech et al showed that, in ICH patients, the early use of a PLT improved platelet activity assay results and was associated ultimately with a smaller hemorrhage size and greater independence at 3 months [22]. However, these results were only analyzed using a 2-sample test for equality of proportions, without multivariate regressions methods.

We conducted this present study to clarify the impact of a PLT on survival of patients with ICH after taking APA.

## Patients and Methods

### Ethics

The Ethics Committee of Kitasato University School of Medicine approved this study (B12–19). Poster presentation for this research was recommended and Informed consent was not obtained from each patient because of the retrospective nature of the study. Patient information was anonymized and de-identified prior to analysis.

### Patients

This retrospective cohort analysis included patients who were newly diagnosed with ICH between January 2006 and June 2011 at the tertiary emergency center of Kitasato University Hospital.

Six types of APAs (aspirin, clopidogrel, ticlopidine, cilostazol, sarpogrelate, and dipyridamole) were being taken by 75 patients. However, only the 66 patients receiving aspirin, clopidogrel, and ticlopidine as an “APA” were basically used in our analyses; patients treated with cilostazol (n = 5) [26], sarpogrelate (n = 1) [27], dipyridamole (n = 3) [28] were excluded, because these three drugs are widely recognized as lower bleeding risk. However, we used all six APAs as a variable in the “broad APA”.

Male and female patients, aged at least 20 years, with a recent ICH diagnosed by computed tomography (CT) scan and no evidence of a traumatic cerebral hemorrhage, were eligible for inclusion in the study. Patients were excluded if they had an ICH with ruptured aneurysm in the brain; bleeding arteriovenous malformations in the brain; a brain tumor; or if the ICH had occurred during pregnancy or just after delivery.

A dedicated multidisciplinary team including attending medical doctors, residents, pharmacists, physiotherapists and nurses, cared for all patients.

### Methods followed at the time of diagnosis

The following clinical data were collected on arrival, and reviewed and analyzed: age; sex; Glasgow Coma Scale (GCS) score on arrival, which is scored between 3 and 15, 3 being the worst, and 15 the best; clinical background; complications before onset of ICH; elapsed time, defined as the duration between estimated onset and arrival at our hospital; administration of ATA including APAs and anticoagulants; and laboratory data on arrival, including prothrombin time (PT), activated partial thromboplastin time (aPTT), platelet count, and fibrinogen. Administration of a PLT after arrival, and outcomes until the seventh day, and 90^th^ day were then recorded. Neurological outcome at 90^th^ day was also collected. Glasgow outcome scale [29] and modified Rankin scale [30] are standard to express the neurological outcome, however, we simplified the neurological outcome scale such as dead, dependently alive, independently alive, because this neurological information was reported from not only healthcare-providers but also non-healthcare-providers such as the patients' families. Elapsed time was defined as the duration from the onset of the ICH to the date of arrival at our center. Observation time was defined as the duration from the date of onset of the ICH to the date of death or to the end of the follow-up.

CT scans were taken on arrival at the center and 24 hours post-arrival and were used to document the initial ICH location (e.g., subcortical, supratentorial or infratentorial hemorrhage), presence or absence of an intraventricular hemorrhage (IVH), and the volume of hemorrhage (volume of hemorrhage was calculated using a previously published method [31]). On the 24-hour follow-up CT scan, cases showing obvious hemorrhage enlargement (≥10% increase in volume compared with on arrival), IVH, or new hemorrhage were judged as showing disease progression.

Primary endpoints were: 1) mortality at 7th and 90th days, and neurological outcome on the 90th day after arrival; and 2) impact of PLT on survival of patients with ICH.

### Statistics

Continuous variables are presented as the median value (25th and 75th percentiles). Relationships between APA and clinical background factors were assessed using the Wilcoxon's test. Numerical variables are provided as N (%). Relationships between APA and clinicopathological parameters were assessed using Pearson's chi-square test or Fisher's exact test, as appropriate.

Multivariate logistic regression analyses and the Cox proportional hazards regression model were used to estimate the independent prognostic effect of APA on outcome within 7 days and within 90 days of arrival by adjusting for age, GCS, liver cirrhosis (LC), hypertension (HT), diabetes mellitus (DM), warfarin, location and volume of ICH, disease progression, laboratory data and transfusions, respectively.

All reported P-values are two-sided. A P-value of 0.05 or less was considered statistically significant. Analyses were performed using SPSS version 17.0 software (SPSS, Chicago, IL).

## Results

### Patient background characteristics, assessment of APA use, and its correlation with clinical parameters

We enrolled 432 patients with ICH (259 men, 60%) into our study; they had a median age on arrival of 67.0 years (range, 40–95 years). The location of the ICH was subcortical in 72 patients (16.7%), supratentorial in 233 (53.9%), and infratentorial in 133 (30.8%) (Tables 1 and 2).

**Table 1:** Patient demographics and background characteristics.

	All patients with ICH.			
	Number (%) patients			
Factors	**Total**	**APA**		
		**+**	**−**	
	432 (100)	66 (15.3)	366 (84.7)	P-value
**Male**	259 (60)	47 (71.2)	212 (57.9)	0.04
**Complication**				
*Chronic Renal Failure*	43 (10)	11 (16.7)	32(8.7)	0.047
*Diabetes mellitus*	55 (12.7)	16 (24.2)	39 (10.7)	0.002
*Hypertension*	388 (89.8)	62 (93.9)	326 (89.1)	0.22
*Liver cirrhosis*	21 (4.9)	1 (1.5)	20 (5.5)	0.17
*Coronary heart disease*	24 (5.6)	15 (22.7)	9 (2.5)	<0.0001
*Transient ischemic attack*	26 (6.0)	14 (21.2)	12 (3.3)	<0.0001
*Atrial fibrillation*	15 (3.5)	6 (9.1)	9 (2.5)	0.007
*Deep vein thrombosis*	4 (0.9)	0	4 (1.1)	0.39
*Valvular disease of heart*	5 (1.2)	1 (1.5)	4 (1.1)	0.77
*Warfarin*	28 (6.5)	10 (15.2)	18 (4.9)	0.002
**Location**				
*Subcortical*	72(16.7)	13 (19.7)	59 (16.1)	0.47
*Supratentorial*	233 (53.9)	34 (51.5)	199 (54.4)	0.67
*Infratentorial*	133 (30.8)	19 (28.8)	114 (31.2)	0.7
**IVH**	113 (26.2)	17 (25.7)	96 (26.2)	0.94
**Operation**	89 (20.6)	5 (7.6)	84 (22.9)	0.004
**Transfusion**				
*Platelet*	16 (3.7)	6 (9.1)	10 (2.7)	0.01
*Fresh frozen plasma*	29 (6.7)	5 (7.6)	24 (6.6)	0.76
**Disease progression**	247 (57.4)	32(49.2)	215 (58.9)	0.15
**Outcome at 7th day**				
*Died*	166 (38.4)	30 (45.5)	136 (37.2)	0.2
**Outcome at 90th day**				0.14
*Died*	178 (41.2)	31 (47)	147 (40.2)	
*Alive (independent)*	65 (15.0)	6 (9.1)	59 (16.1)	
*Alive (dependent)*	63 (14.6)	6 (9.1)	57 (15.6)	
*Lost of follow up*	126 (29.2)	23 (34.8)	103 (28.1)	

Numerical variates are given as N (%) and are compared with the

chi-square test or Fisher's exact test, as appropriate.

N.D.:not done.

ICH: intracerebral hemorrhage. APA: anti-platelet agents.

IVH: intraventricular hemorrhage.

**Table 2:** Patient characteristics.

		Patients with ICH			
Variables			Anti-platelet agents		
			+	−	
	N	432 (100)	66 (15.3)	366 (84.7)	P-value
**Age**	432	67 (57, 75)	74 (64, 78)	65 (56,74)	<0.0001
**Observation time(day)**	432	33.5 (2.0, 151.3)	11 (2, 104.5)	38.5 (2, 174)	0.13
**Elapsed time(hour)**	431	2 (1, 3)	1.5 (1, 2.3)	2 (1,3)	0.53
**Glasgow Coma Scale**	432	6 (4,13)	7 (4,13)	6.5 (4,13)	0.92
**Volume of hemorrhage**	427	68 (27, 168)	83 (34.8, 185.5)	65.3 (26.8, 168)	0.39
**Laboratory data**					
***Platelet (10^4^/µL)***	427	19.6(15.6, 24.7)	18.7 (16.0, 22.1)	19.7 (15.5, 24.9)	0.42
***Prothrombin time (%)***	423	97 (86, 103)	95 (80.5, 100.3)	98 (88, 105)	0.01
***aPTT (sec)***	422	31.1 (27.9, 34.6)	33.2 (29.3, 36.8)	30.8 (27.7, 34.4)	0.008
***Fibrinogen (mg/dL)***	419	347 (291, 413)	371 (313.5, 463)	339.5 (289,402.5)	0.01

Continous variables are presented as median (25, 75 percentile) and are compared using the Wilcoxon's test.

Elasped time: time at arrival at emergency treatment center minus estimated onset time.

aPTT: activated partial thromboplastin time. APA: anti-platelet agents.

Of these patients, 66 (15.7%) had been prescribed APA prior to ICH onset, comprising aspirin (n = 50, 75%), aspirin and clopidogrel (n = 12, 18.2%), or clopidogrel or ticlopidine (each n = 2, 3.0%) (Table 3).

**Table 3:** Details of anti-platelet agents.

Agents	Number (%) patients
	(N = 66)
Aspirin only	50 (75.0)
Aspirin + clopidogrel	12(18.2)
Clopidogrel only	2 (3.0)
Ticlopidine	2 (3.0)

Numerical variables are given as N (%).

Regarding the reasons for APA administration, there was a statistically significantly greater incidence of DM (P = 0.047), coronary heart disease (CHD) (P<0.0001), and transient ischemic attack (TIA) (P<0.0001) among patients taking APAs compared with patients who were not (Table 1). PLT and fresh frozen plasma (FFP) transfusion were administered to 16 (3.7%) and 29 (6.7%) patients, respectively. The decision to administer a PLT to patients also taking an APA was made by the attending doctors, who may have been concerned about bleeding tendencies due to APA. On the other hand, PLT was also given to patients without APA because of lower platelet counts (<1×10^5^/µL) (n = 6, range of platelet value; 4.0∼9.6×10^4^/µL, mean platelet value; 7.6×10^4^/µL) according to CNS guideline[32], coagulopathy because of warfarin and CRF (n = 2) and unknown reason (n = 2). By day 7 and by day 30, respectively, 166 (38.4%) and 178 (41.2%) patients had died due to an ICH (Table 1).

### Characteristics of patients taking APAs, assessment of platelet transfusions received, and their correlation with clinical parameters (Tables 4 and 5)

**Table 5:** Patient Characteristics.

		Patients with ICH and APA		
Variables		Platelet transfusion		
		+	-	
	N	6 (9.1)	60 (90.9)	P-value
**Age**	66	73.5 (66, 76.3)	74 (64, 78)	0.74
**Observation time(day)**	66	193 (82.8, 1257)	7.5 (2, 78.5)	0.006
**Elapsed time(hour)**	66	1.5 (1, 2.75)	1.5 (1, 2.75)	0.84
**Glasgow Coma Scale**	66	12 (6.8,12)	6 (4,12)	0.07
**Volume of hemorrhage**	65	87.5 (50,150.8)	83 (32.2, 198)	0.91
**Laboratory data**				
***Platelet (10^4^/µL)***	65	19.3 (14.9,27.3)	18.7 (16, 21.9)	0.85
***Prothrombin time (%)***	66	80 (50, 91.5)	95 (82.3, 100.8)	0.14
***aPTT (sec)***	66	35.4 (27.1, 36.6)	33.1 (29.5, 37.2)	0.86
***Fibrinogen (mg/dL)***	65	330.5 (253.5, 432.5)	371 (314, 463)	0.25

Continous variables are presented as median (25, 75 percentile) and are compared using the Wilcoxon's test.

Elasped time: time at arrival at emergency treatment center minus estimated onset time.

aPTT: activated partial thromboplastin time. APA: anti-platelet agents.

66 patients with an ICH (47 men, 71.2%), with a median age on arrival of 74.0 years, had previously received an APA and were grouped according to whether or not they subsequently received a PLT. No statistically significant differences were observed between two groups with respect to their baseline characteristics. There were no deaths among the 6 patients who had taken an APA and received a PLT compared with 30 deaths among the 60 patients in the ‘APA with no PLT’ group (P = 0.03; Table 4). Thus, PLT seemed to be an effective treatment for patients with ICH who were taking an APA. And APA with PLT patients seemed to show better neurological outcome at 90^th^ day, although it was difficult to assess the neurological outcome because 34.8% patients were lost to follow up.

**Table 4:** Patient demographics and background characteristics.

	Patients with APA.			
	Number (%) patients			
Factors	**Total**	**platelet transfusion**		
		**+**	**−**	
	66 (100)	6 (9.1)	60 (90.9)	P-value
**Male**	47 (71.2)	5 (83)	42 (70)	0.49
**Complication**				
*Chronic Renal Failure*	11 (16.7)	0	11(18.3)	0.25
*Diabetes mellitus*	16 (24.2)	1 (16.7)	15 (25.0)	0.65
*Hypertension*	62 (93.9)	5 (83.3)	57 (95.0)	0.32
*Liver cirrhosis*	1 (1.5)	0	1(1.7)	0.75
*Coronary heart disease*	15 (22.7)	3 (50)	12 (20)	0.09
*Transient ischemic attack*	14 (21.2)	1(16.7)	13 (21.7)	1.00
*Atrial fibrillation*	6 (9.1)	1(16.7)	5 (8.3)	0.45
*Deep vein thrombosis*	0	0	0	N.D.
*Valvular disease of heart*	1 (1.5)	0	1 (1.7)	1.00
*Warfarin*	10 (15.2)	2 (33.3)	8 (13.3)	0.33
**Location**				
*Subcortical*	13 (19.7)	1 (25)	12 (20.0)	1.00
*Supratentorial*	34(51.5)	2 (33.3)	32 (53.3)	0.42
*Infratentorial*	19 (28.8)	3 (50)	16 (26.7)	0.34
**IVH**	17 (25.8)	2 (33.3)	15 (25.0)	0.64
**Operation**	5 (7.6)	3 (50)	2 (3.3)	0.0001
**Transfusion**				
*Platelet*	6 (9.1)	6 (100)	60 (90.9)	0.64
*Fresh frozen plasma*	5 (7.6)	2 (33.3)	3 (5.0)	0.06
**Disease progression**	32 (49.2)	3 (50)	29 (49.2)	1.00
**Outcome at 7th day**				
*Died*	30 (45.5)	0	30 (50)	0.03
**Outcome at 90th day**				0.008
*Died*	31 (47)	0	31 (77.5)	
*Alive (independent)*	6 (14)	2 (66.7)	4 (10)	
*Alive (dependent)*	6 (14)	1 (33.3)	5 (12.5)	
*Lost of follow up*	23 (34.8)	3 (50)	20 (33.3)	

Numerical variates are given as N (%) and are compared with the

chi-square test or Fisher's exact test, as appropriate.

N.D.:not done.

ICH: intracerebral hemorrhage. APA: anti-platelet agents.

IVH: intraventricular hemorrhage.

### Association between APA and mortality at the 7th day after arrival (Table 6) and at the 90^th^ day (Table 7)

Before multivariate analysis, a correlation coefficient matrix of variables was made, and the Kendall tau correlation coefficients between variables were less than 0.6, indicating no strong multicollinearity among our selected variables.

Variables were included in the multivariate logistic regression analysis using the forced entry method. Multivariate logistic regression analysis indicated APA (odds ratio (OR), 5.12; 95% confidence interval (CI), 1.59–16.5; P = 0.006), lower GCS (OR, 0.64; 95% CI, 0.57–0.74; P<0.0001), and disease progression (OR, 32; 95% CI, 10.24–99.7; P<0.0001) as independent risk factors for death by day 7 (Table 6, Model 1). Furthermore, when we used the category “broad APA” as a variable in the analysis instead of APA, the OR decreased to 2.7 from 5.12 of APA (Table 6, Model 5).

**Table 6:** Impact of platelet transfusion and patient factors on the mortality at day 7.

					Model 1				Model 2				Model 3				Model 4				Model 5			
					Effect of APA on survival				Effect of FFP and PLT on survival				Effect of FFP on survival				Effect of PLT on survival				Effect of broad APA on survival			
	Univariate				Multivariate				Multivariate				Multivariate				Multivariate				Multivariate			
Variables	B	OR	P	95% CI	B	OR	P	95% CI	B	OR	P	95% CI	B	OR	P	95% CI	B	OR	P	95% CI	B	OR	P	95% CI
Age	0.01	1.01	0.19	0.99-1.03	0.03	1.03	0.1	1.00-1.06	0.02	1.02	0.22	0.99-1.05	0.02	1.02	0.2	0.98-1.05	0.03	1.03	0.1	1.00-1.06	0.03	1.03	0.04	1.00-1.06
Sex	0.03	1.01	0.67	0.72-1.69																				
Elapsed time	-0.01	0.98	0.29	0.96-1.01																				
Glasgow coma scale	-0.55	0.57	<0.0001	0.50-0.64	-0.44	0.64	<0.0001	0.57-0.74	-0.43	0.66	<0.0001	0.57-0.75	-0.43	0.65	<0.0001	0.57-0.74	-0.43	0.65	<0.0001	0.57-0.74	-0.44	0.65	<0.0001	0.57-0.73
Chronic Renal Failure	0.47	1.61	0.14	0.85-3.02																				
Liver cirrhosis	1.22	3.3	0.02	1.30-8.56	0.88	2.4	0.24	0.55-10.5	0.92	2.5	0.25	0.52-12.0	0.9	2.47	0.25	0.53-11.4	0.9	2.47	0.24	0.54-11.2	0.82	2.27	0.28	0.52-9.86
Hypertension	-0.13	0.88	0.72	0.42-1.82	-0.71	0.43	0.28	0.14-1.77	-0.59	0.56	0.39	0.15-2.14	-0.65	0.52	0.34	0.14-1.99	-0.56	0.4	0.57	0.15-2.11	-0.65	0.52	0.31	0.15-1.83
CHD	0.35	1.41	0.48	0.54-3.67																				
Diabetes mellitus	-0.58	0.56	0.12	0.27-1.15	-0.56	0.57	0.30	0.20-1.66	-0.6	0.55	0.29	0.18-1.66	-0.6	0.55	0.29	0.18-1.67	-0.63	0.53	0.18	0.12-1.57	-0.48	0.62	0.37	0.22-1.76
Atrial fibrillation	-1.05	0.34	0.33	0.04-2.95																				
TIA	0.47	1.60	0.32	0.63-4.05																				
Anti-platelet agents	0.17	1.18	0.61	0.62-2.24	1.63	5.12	0.006	1.59-16.5	1.91	6.78	0.004	1.84-24.9	1.78	5.91	0.006	1.68-20.9	1.86	6.48	0.003	1.88-22.3				
Warfarin	0.35	1.42	0.37	0.66-3.07	-0.28	0.76	0.7	0.18-3.1	0.37	1.44	0.67	0.27-7.68	0.25	1.28	0.77	0.25-6.51	0.03	1.03	0.97	0.23-4.71	-0.19	0.83	0.8	0.2-3.4
Broad APA	0.15	1.16	0.57	0.70-1.92																	0.99	2.7	0.06	0.95-7.63
Location																								
subcortical	-0.24	0.79	0.43	0.44-1.43																				
supratentorial	-0.33	0.72	0.14	0.47-1.11																				
infratentorial	0.73	2.01	0.002	1.31-3.28	0.23	1.26	0.53	0.61-2.6	0.36	1.42	0.36	0.67-3.03	0.37	1.28	0.76	0.68-3.07	0.23	1.25	0.55	0.60-2.61	0.24	1.27	0.52	0.62-2.60
Volume of ICH	0.003	1.01	<0.0001	1.006-1.01	0.003	1.003	0.06	1.00-1.01	0.004	1.004	0.04	1-1.01	0.004	1.002	0.04	1.00-1.01	0.003	1.003	0.046	1-1.007	0.003	1.003	0.06	1-1.006
Disease progression	3.96	52.5	<0.0001	20.6-133.9	3.46	32	<0.0001	10.24-99.7	3.5	33.1	<0.0001	10.1-108.1	3.47	32.2	<0.0001	10.1-102.6	3.51	33.4	<0.0001	10.4-107.7	3.28	26.6	<0.0001	9.18-77.2
Operation	0.63	1.89	<0.0001	1.39-2.54																				
IVH	0.20	1.23	0.40	0.76-1.97																				
Platelet count	-0.04	0.96	0.011	0.93-0.99	-0.006	0.98	0.47	0.93-1.03	-0.04	0.97	0.21	0.91-1.02	-0.03	0.97	0.22	0.92-1.02	-0.03	0.97	0.32	0.92-1.03	-0.02	0.98	0.32	0.93-1.03
PT	-0.02	0.99	0.001	0.98-0.99																				
aPTT	0.04	1.02	0.11	0.99-1.05																				
Fibrinogen	-0.001	1.00	0.52	0.99-1.00	0.001	1	0.8	0.93-1.03	0.001	1	0.98	1.00-1.01	0.0001	1	0.98	0.997-1.003	0.0001	1	0.91	0.997-1.003	0.001	1.001	0.72	0.998-1.003
Platelet transfusion	-0.98	4.02	0.07	0.90-18.1					-1.49	0.23	0.26	0.02-3.02					-2.62	0.03	0.07	0.01-0.75				
FFP transfusion	1.44	4.20	0.009	1.44-12.3					2.23	0.04	0.008	1.81-48.0	2.59	13.3	0.001	2.85-62.6								

CHD: coronary heart disease; PH of TIA: past history of transient ischemic attack; APA: anti-platelet agents; IVH: intraventricular hemorrhage.

ICH: intracerebral hemorrhage; PT: prothrombin time; aPTT: activated partial thromboplastin time; OR: odds ratio; CI: confidence interval.

By way of precaution, we tried both the forward and backward methods for selecting variables after our analyses, and GCS, APA, and disease progression were all selected by both methods.

By cox proportional hazards regression analysis, APA was the independent risk factor for the survival at the 90^th^ day (hazard ratio, 1.87; 95% CI, 1.20–2.91; P = 0.006) (Table 7).

**Table 7:** Impact of platelet transfusion and patient factors on the mortality at day 90.

					Model 1				Model 2		
					Effect of APA on survival				Effect of PLT on survival		
		Univariate				Multivariate				Multivariate	
Variables	B	HR	P	95% CI	B	HR	P	95% CI	B	HR	P
**Age**	0.01	1.01	0.19	0.99–1.02	0.01	1.01	0.1	0.99–1.02	0.01	1.01	0.22
**Sex**	0.07	1.01	0.67	0.79–1.45							
**Elapsed time**	−0.02	0.98	0.10	0.96–1.004							
**Glasgow coma scale**	−0.38	0.68	<0.0001	0.64–0.73	−0.28	0.76	<0.0001	0.69–0.82	−0.27	0.77	<0.0001
**Chronic Renal Failure**	0.28	1.33	0.21	0.85–2.08							
**Liver cirrhosis**	0.85	2.33	0.002	1.37–3.95	0.51	1.7	0.13	0.86–3.22	0.59	1.8	0.25
**Hypertension**	−0.09	0.91	0.71	0.57–1.49	−0.26	0.77	0.35	0.45–1.33	−0.21	0.81	0.39
**CHD**	0.35	1.41	0.51	0.48–3.67							
**Diabetes mellitus**	−0.15	0.86	0.52	0.55–1.36	−0.15	0.86	0.58	0.51–1.47	−0.17	0.84	0.29
**Atrial fibrillation**	−1.05	0.34	0.33	0.04–1.95							
**TIA**	0.47	1.60	0.32	0.63–3.04							
**Anti-platelet agents**	0.21	1.24	0.28	0.84–1.82	0.62	1.87	0.006	1.20–2.91	0.64	1.9	0.004
**Warfarin**	0.26	1.30	0.37	0.74–2.28	0.1	0.76	0.76	0.59–2.04	0.21	1.24	0.67
**Location**											
*subcortical*	−0.13	0.88	0.54	0.59–1.32							
*supratentorial*	−0.26	0.77	0.08	0.57–1.04							
*infratentorial*	0.48	1.61	0.002	1.19–2.18	0.08	1.08	0.69	0.74–1.56	0.06	1.07	0.36
**Volume of ICH**	0.004	1.004	<0.0001	1.003–1.005	0.001	1.001	0.06	1.00–1.003	0.001	1.001	0.06
**Disease progression**	2.94	18.9	<0.0001	9.98–35.95	2.43	11.3	<0.0001	4.84–26.6	2.4	11.5	<0.0001
**Operation**	0.63	1.89	<0.0001	1.39–2.54							
**IVH**	0.20	1.23	0.40	0.76–1.97							
**Platelet count**	−0.04	0.96	0.001	0.94–0.99	0.001	1.001	0.95	0.98–1.03	−0.001	0.97	0.21
**PT**	−0.009	0.99	0.002	0.98–0.99							
**aPTT**	0.001	1.02	0.99	0.99–1.01							
**Fibrinogen**	−0.001	1.00	0.29	0.99–1.001	0.001	1	0.81	0.99–1.002	0.001	1	0.98
**Platelet transfusion**	−0.97	0.38	0.09	0.12–1.18					−1.74	0.18	0.09
**FFP transfusion**	1.08	2.96	0.02	1.21−7.18							

CHD: coronary heart disease; TIA: transient ischemic attack; APA: anti-platelet agents; IVH: intraventricular hemorrhage.

ICH: intracerebral hemorrhage; PT: prothrombin time; aPTT: activated partial thromboplastin time; HR:hazard ratio; CI: confidence interval.

### Impact of PLT on survival within 7 days after arrival of those patients with an ICH, who were also taking an APA

Administrations of a PLT and/or FFP were added as a variable to our model (Table 6, Models 2–4). The use of APA was still an independent prognostic factor in these analyses, and PLT seemed to be beneficial to the survival of ICH patients (Model 2, 4), regardless of the use of APA. The OR of APA increased from 5.12 (95% CI, 1.59–16.5; P = 0.006) in the Model 1 to 6.48 (95% CI, 1.88–22.3; P = 0.003) in the Model 4 because of the small number of events, an assumption that was drawn from the widening of the 95% CI.

## Discussion

We assessed the effects of prior administration of APA on patients with ICH, which yielded several important results. First, we confirmed that APA worsened survival in patients with ICH within 7 days and 90 days after arrival at the emergency center. Second, giving a PLT to ICH patients who were taking an APA was a favorable factor for survival.

First, to discuss our finding that taking an APA worsened survival in patients with an ICH within 7 days after arrival, two possibilities may explain this result: 1) the mode of action of APAs; and 2) the underlying disease that had been controlled by APA therapy.

APAs inhibit the activation and aggregation of platelets after vascular injury, and thus induce a bleeding tendency, so we can easily imagine disease progression after the onset of ICH in patients taking APAs. According to the Bleeding with Anti-Thrombotic therapy (BAT) study, incidences of ICH reported in cases administered a single APA, two types of APA, warfarin only, or APA with warfarin were 0.34%, 0.60%, 0.62%, and 0.96%, respectively [33]. In our study, 50 of 66 patients had been prescribed a single APA, and 12 had been prescribed two APAs. For patients with warfarin, measuring PT regularly is an efficient and very easy way to prescribe the proper dose of warfarin. Furthermore, treatment strategies for patients with an ICH who are also taking warfarin have been largely established, and include the use of vitamin K, FFP, and prothrombin complex concentrates [34], [35], [36]. For patients with an ICH who are taking APA, treatment strategies to recover platelet function have not been established, yet.

In terms of the second possibility, patients who need APA have atherosclerotic diseases such as CHD or TIA, which are considered as risk factors for survival of ICH. Among our patients receiving APA, 15 patients (22.7%) had CHD and 14 (21.2%) had experienced a TIA.

Second, to discuss our finding that giving a PLT to ICH patients taking APA was a favorable factor for survival (Table 4). In previous studies, the effects of PLT on spontaneous ICH cases taking APAs were unclear. We suggest that the key to solving this uncertainty is to establish when a PLT should be given and to whom.

To address the first question of timing of administration of the PLT, which is important to stop hematoma expansion (HE). Naidech reported [22] that administration of a PLT within 12 hours led to good neurological results; unfortunately, their results were not analyzed by multivariate regression. Several studies have indicated that early HE occurs in 18–38% of patients scanned within 3 hours of ICH onset, and more than 70% develop at least some degree of HE within 24 hours of symptom onset, even in the absence of known coagulopathy, suggesting an active bleeding process in the hyper acute phase of ICH [37]. Unfortunately, we could not check the actual timing of PLT administration, although it appeared to be more than 6 hours after arrival. The median elapsed time from onset was 2 hours (Table 2). After arrival at our hospital, checking neurological symptoms, eliciting the patient's medical and drug histories from the family, laboratory examinations, and a CT examination were performed simultaneously. As a result, in our hospital, at least 6 hours may have elapsed before considering and ordering PLT.

Secondly, to address the question of which patients truly need a PLT after developing an ICH while taking APAs. The negative impact of unnecessary administration of PLT includes over-coagulation and effects of the cytokines associated with the PLT will be concerned. After ICH onset, decreased blood flow to the area surrounding the clot causes local neuronal ischemia, leading to further cytotoxic edema and the toxic release of excitatory amino acids and inflammatory mediators [38]. On diffusion-weighted imaging, a significant number of ICH patients show acute ischemic lesions that are not contiguous with the hematoma [39], [40]. A PLT may induce unnecessary thrombus in and around the ischemic area, resulting in enlargement of the ischemic lesion and worsening of the brain injuries.

The ICH area shows thrombin-induced activation of the inflammatory cascade [41] and overexpression of matrix metalloproteinase (MMPs), representing additional mechanisms contributing to breakdown of the blood-brain barrier, brain edema growth and neuronal death, all of which are recognized as secondary brain injuries after the onset of ICH [42]. Platelets are the major supplier of MMPs [43], and therefore brain injury may be worsened by a PLT, as it will provide an inflammatory stimulus to the injured brain.

Aspirin resistance is also a well-known phenomenon [15], [20], and has been well observed in Japan [44]. Furthermore, 60% of Japanese are low responders to clopidogrel because of a CYP2C19 polymorphism [19], [45]. However, easy, fast, and low-cost laboratory examinations to monitor the effect of APA on an individual patient's platelets are not readily available. For some patients, a certain dose may not be sufficient to prevent thrombotic events, while for others, that same dose might cause dangerous bleeding complications such as ICH. For low responders to APA, in whom platelet function and coagulation parameters are normal, even when given concomitantly with APA, PLT might induce over-coagulation around the ICH site.

Taking drug resistance into consideration, clarification of the effects of PLT on ICH prognosis will require a prospective cohort study with monitoring of the effects of APA.

A key limitation in this study was the short duration of observation, because patients were often moved to other institutions within a short period of time following admission, due to limited capacity at our hospital. In addition, we need an increased sample size to evaluate better the PLT effect in the multivariate regression analysis. Another disadvantage in this study was that the decision to give a PLT depended on the resident neurosurgeon. However, the strength of our study was that this was the first to look at the effect of PLT in a population of Asian patients with ICH plus concomitant APA; all three previous studies have dealt mainly with Caucasian patients [10], [22], [23]. As Asian people tend to be APA resistant, PLT seemed to be less necessary compared with Caucasians even in cases of ICH with APA. Finally, as this was a single-institution study, our patients were evaluated using consistent methods and procedures.

To evaluate the true effects of PLT on ICH survival, animal models and prospective stratified cohorts taking into account the effects of APAs are necessary.

## References

[1] Hermosillo AJ, Spinler SA (2008) Aspirin, clopidogrel, and warfarin: is the combination appropriate and effective or inappropriate and too dangerous? Ann Pharmacother 42: 790–805. 18477734 10.1345/aph.1K591

[2] Dewilde WJ, Oirbans T, Verheugt FW, Kelder JC, De Smet BJ, et al (2013) Use of clopidogrel with or without aspirin in patients taking oral anticoagulant therapy and undergoing percutaneous coronary intervention: an open-label, randomised, controlled trial. Lancet 381: 1107–1115. 23415013 10.1016/S0140-6736(12)62177-1

[3] Steinhubl SR, Bhatt DL, Brennan DM, Montalescot G, Hankey GJ, et al (2009) Aspirin to prevent cardiovascular disease: the association of aspirin dose and clopidogrel with thrombosis and bleeding. Ann Intern Med 150: 379–386. 19293071 10.7326/0003-4819-150-6-200903170-00006

[4] Hennekens CH, Kaski JC (2013) Secondary prevention of cardiovascular disease. In: Cannon CP, editor. Up To Date. Available: http://www.uptodate.com/contents/secondary-prevention-of-cardiovascular-disease. Accessed 2014 May 7.

[5] Kiyohara Y, Kubo M, Kato I, Tanizaki Y, Tanaka K, et al (2003) Ten-year prognosis of stroke and risk factors for death in a Japanese community: the Hisayama study. Stroke 34: 2343–2347. 14500930 10.1161/01.STR.0000091845.14833.43

[6] Tanaka H, Ueda Y, Date C, Baba T, Yamashita H, et al (1981) Incidence of stroke in Shibata, Japan: 1976-1978. Stroke 12: 460–466. 7314167 10.1161/01.str.12.4.460

[7] Sacco S, Marini C, Toni D, Olivieri L, Carolei A (2009) Incidence and 10-year survival of intracerebral hemorrhage in a population-based registry. Stroke 40: 394–399. 19038914 10.1161/STROKEAHA.108.523209

[8] Rincon F, Mayer SA (2013) The epidemiology of intracerebral hemorrhage in the United States from 1979 to 2008. Neurocrit Care 19: 95–102. 23099848 10.1007/s12028-012-9793-y

[9] Hankey GJ, Sudlow CL, Dunbabin DW (2000) Thienopyridines or aspirin to prevent stroke and other serious vascular events in patients at high risk of vascular disease? A systematic review of the evidence from randomized trials. Stroke 31: 1779–1784. 10884487 10.1161/01.str.31.7.1779

[10] Creutzfeldt CJ, Weinstein JR, Longstreth WT Jr, Becker KJ, McPharlin TO, et al (2009) Prior antiplatelet therapy, platelet infusion therapy, and outcome after intracerebral hemorrhage. J Stroke Cerebrovasc Dis 18: 221–228. 19426894 10.1016/j.jstrokecerebrovasdis.2008.10.007 PMC2830090

[11] Roquer J, Rodriguez Campello A, Gomis M, Ois A, Puente V, et al (2005) Previous antiplatelet therapy is an independent predictor of 30-day mortality after spontaneous supratentorial intracerebral hemorrhage. J Neurol 252: 412–416. 15739042 10.1007/s00415-005-0659-5

[12] Toyoda K, Okada Y, Minematsu K, Kamouchi M, Fujimoto S, et al (2005) Antiplatelet therapy contributes to acute deterioration of intracerebral hemorrhage. Neurology 65: 1000–1004. 16217049 10.1212/01.wnl.0000179178.37713.69

[13] Thompson BB, Bejot Y, Caso V, Castillo J, Christensen H, et al (2010) Prior antiplatelet therapy and outcome following intracerebral hemorrhage: a systematic review. Neurology 75: 1333–1342. 20826714 10.1212/WNL.0b013e3181f735e5 PMC3013483

[14] Hennekens C, Cutlip D, Zehnder J (2013) Nonresponse and resistance to aspirin. In: Leung L, editor. PA: Wolters Kluwer.

[15] Fitzgerald R, Pirmohamed M (2011) Aspirin resistance: effect of clinical, biochemical and genetic factors. Pharmacol Ther 130: 213–225. 21295071 10.1016/j.pharmthera.2011.01.011

[16] Wijeyeratne YD, Heptinstall S (2011) Anti-platelet therapy: ADP receptor antagonists. Br J Clin Pharmacol 72: 647–657. 21518389 10.1111/j.1365-2125.2011.03999.x PMC3187865

[17] Sarafoff N, Byrne RA, Sibbing D (2012) Clinical use of clopidogrel. Curr Pharm Des 18: 5224–5239. 22724411 10.2174/138161212803251853

[18] Flores-Runk P, Raasch RH (1993) Ticlopidine and antiplatelet therapy. Ann Pharmacother 27: 1090–1098. 8219445 10.1177/106002809302700915

[19] Jinnai T, Horiuchi H, Makiyama T, Tazaki J, Tada T, et al (2009) Impact of CYP2C19 polymorphisms on the antiplatelet effect of clopidogrel in an actual clinical setting in Japan. Circ J 73: 1498–1503. 19531897 10.1253/circj.cj-09-0019

[20] Eikelboom JW, Hirsh J, Weitz JI, Johnston M, Yi Q, et al (2002) Aspirin-resistant thromboxane biosynthesis and the risk of myocardial infarction, stroke, or cardiovascular death in patients at high risk for cardiovascular events. Circulation 105: 1650–1655. 11940542 10.1161/01.cir.0000013777.21160.07

[21] Washington CW, Schuerer DJ, Grubb RL Jr (2011) Platelet transfusion: an unnecessary risk for mild traumatic brain injury patients on antiplatelet therapy. J Trauma 71: 358–363. 21825939 10.1097/TA.0b013e318220ad7e

[22] Naidech AM, Liebling SM, Rosenberg NF, Lindholm PF, Bernstein RA, et al (2012) Early platelet transfusion improves platelet activity and may improve outcomes after intracerebral hemorrhage. Neurocrit Care 16: 82–87. 21837536 10.1007/s12028-011-9619-3 PMC6211165

[23] Ducruet AF, Hickman ZL, Zacharia BE, Grobelny BT, DeRosa PA, et al (2010) Impact of platelet transfusion on hematoma expansion in patients receiving antiplatelet agents before intracerebral hemorrhage. Neurol Res 32: 706–710. 20819399 10.1179/174313209X459129

[24] Naidech AM, Jovanovic B, Liebling S, Garg RK, Bassin SL, et al (2009) Reduced platelet activity is associated with early clot growth and worse 3-month outcome after intracerebral hemorrhage. Stroke 40: 2398–2401. 19443791 10.1161/STROKEAHA.109.550939

[25] Batchelor JS, Grayson A (2012) A meta-analysis to determine the effect on survival of platelet transfusions in patients with either spontaneous or traumatic antiplatelet medication-associated intracranial haemorrhage. BMJ Open 2: e000588. 10.1136/bmjopen-2011-000588 PMC3326637 22492383

[26] Shinohara Y, Katayama Y, Uchiyama S, Yamaguchi T, Handa S, et al (2010) Cilostazol for prevention of secondary stroke (CSPS 2): an aspirin-controlled, double-blind, randomised non-inferiority trial. Lancet Neurol 9: 959–968. 20833591 10.1016/S1474-4422(10)70198-8

[27] Shinohara Y, Nishimaru K, Sawada T, Terashi A, Handa S, et al (2008) Sarpogrelate-Aspirin Comparative Clinical Study for Efficacy and Safety in Secondary Prevention of Cerebral Infarction (S-ACCESS): A randomized, double-blind, aspirin-controlled trial. Stroke 39: 1827–1833. 18388340 10.1161/STROKEAHA.107.505131

[28] De Schryver EL, Algra A, van Gijn J (2010) Dipyridamole for preventing stroke and other vascular events in patients with vascular disease. Cochrane Database Syst Rev: CD001820. 10.1002/14651858.CD001820 12535415

[29] Jennett B, Snoek J, Bond MR, Brooks N (1981) Disability after severe head injury: observations on the use of the Glasgow Outcome Scale. J Neurol Neurosurg Psychiatry 44: 285–293. 6453957 10.1136/jnnp.44.4.285 PMC490949

[30] van Swieten JC, Koudstaal PJ, Visser MC, Schouten HJ, van Gijn J (1988) Interobserver agreement for the assessment of handicap in stroke patients. Stroke 19: 604–607. 3363593 10.1161/01.str.19.5.604

[31] Kothari RU, Brott T, Broderick JP, Barsan WG, Sauerbeck LR, et al (1996) The ABCs of measuring intracerebral hemorrhage volumes. Stroke 27: 1304–1305. 8711791 10.1161/01.str.27.8.1304

[32] Samama CM, Djoudi R, Lecompte T, Nathan-Denizot N, Schved JF (2005) Perioperative platelet transfusion: recommendations of the Agence Francaise de Securite Sanitaire des Produits de Sante (AFSSaPS) 2003. Can J Anaesth 52: 30–37. 15625253 10.1007/BF03018577

[33] Toyoda K, Yasaka M, Iwade K, Nagata K, Koretsune Y, et al (2008) Dual antithrombotic therapy increases severe bleeding events in patients with stroke and cardiovascular disease: a prospective, multicenter, observational study. Stroke 39: 1740–1745. 18388341 10.1161/STROKEAHA.107.504993

[34] Butler AC, Tait RC (1998) Management of oral anticoagulant-induced intracranial haemorrhage. Blood Rev 12: 35–44. 9597196 10.1016/s0268-960x(98)90028-5

[35] Flaherty ML (2010) Anticoagulant-associated intracerebral hemorrhage. Semin Neurol 30: 565–572. 21207349 10.1055/s-0030-1268866

[36] Flibotte JJ, Hagan N, O'Donnell J, Greenberg SM, Rosand J (2004) Warfarin, hematoma expansion, and outcome of intracerebral hemorrhage. Neurology 63: 1059–1064. 15452298 10.1212/01.wnl.0000138428.40673.83

[37] Balami JS, Buchan AM (2012) Complications of intracerebral haemorrhage. Lancet Neurol 11: 101–118. 22172625 10.1016/S1474-4422(11)70264-2

[38] Dearden NM (1998) Mechanisms and prevention of secondary brain damage during intensive care. Clin Neuropathol 17: 221–228. 9707338

[39] Menon RS, Burgess RE, Wing JJ, Gibbons MC, Shara NM, et al (2012) Predictors of highly prevalent brain ischemia in intracerebral hemorrhage. Ann Neurol 71: 199–205. 22367992 10.1002/ana.22668 PMC3298034

[40] Garg RK, Liebling SM, Maas MB, Nemeth AJ, Russell EJ, et al (2012) Blood pressure reduction, decreased diffusion on MRI, and outcomes after intracerebral hemorrhage. Stroke 43: 67–71. 21980211 10.1161/STROKEAHA.111.629493 PMC3246540

[41] Lee KR, Kawai N, Kim S, Sagher O, Hoff JT (1997) Mechanisms of edema formation after intracerebral hemorrhage: effects of thrombin on cerebral blood flow, blood-brain barrier permeability, and cell survival in a rat model. J Neurosurg 86: 272–278. 9010429 10.3171/jns.1997.86.2.0272

[42] Florczak-Rzepka M, Grond-Ginsbach C, Montaner J, Steiner T (2012) Matrix metalloproteinases in human spontaneous intracerebral hemorrhage: an update. Cerebrovasc Dis 34: 249–262. 23052179 10.1159/000341686

[43] Seizer P, May AE (2013) Platelets and matrix metalloproteinases. Thromb Haemost 110. 10.1160/TH13-02-0113 23864155

[44] Miyata S, Miyata T, Kada A, Nagatsuka K (2008) [Aspirin resistance]. Brain Nerve 60: 1357–1364. 19069170

[45] Hoshino K, Horiuchi H, Tada T, Tazaki J, Nishi E, et al (2009) Clopidogrel resistance in Japanese patients scheduled for percutaneous coronary intervention. Circ J 73: 336–342. 19106460 10.1253/circj.cj-08-0559

